# Ocean pH fluctuations affect mussel larvae at key developmental transitions

**DOI:** 10.1098/rspb.2018.2381

**Published:** 2018-12-19

**Authors:** L. Kapsenberg, A. Miglioli, M. C. Bitter, E. Tambutté, R. Dumollard, J.-P. Gattuso

**Affiliations:** 1 Laboratoire d'Océanographie de Villefranche, Sorbonne Université, CNRS, 181 chemin du Lazaret, 06230 Villefranche-sur-mer, France; 2 Laboratoire de Biologie du Développement de Villefranche, Sorbonne Université, CNRS, 181 chemin du Lazaret, 06230 Villefranche-sur-mer, France; 3 Dipartimento di Scienze della Terra, dell'Ambiente e della Vita, DISTAV, Università di Genova, Genova, Italy; 4 Department of Ecology and Evolution, University of Chicago, Chicago, IL, USA; 5 Marine Biology Department, Centre Scientifique de Monaco, 8 Quai Antoine Ier, MC98000, Monaco, Monaco; 6 Institute for Sustainable Development and International Relations, Sciences Po, 27 rue Saint Guillaume, 75007 Paris, France

**Keywords:** ocean acidification, pH fluctuations, mollusc, development, shell field, trochophore

## Abstract

Coastal marine ecosystems experience dynamic fluctuations in seawater carbonate chemistry. The importance of this variation in the context of ocean acidification requires knowing what aspect of variability biological processes respond to. We conducted four experiments (ranging from 3 to 22 days) with different variability regimes (pH_T_ 7.4–8.1) assessing the impact of diel fluctuations in carbonate chemistry on the early development of the mussel *Mytilus galloprovincialis*. Larval shell growth was consistently correlated to mean exposures, regardless of variability regimes, indicating that calcification responds instantaneously to seawater chemistry. Larval development was impacted by timing of exposure, revealing sensitivity of two developmental processes: development of the shell field, and transition from the first to the second larval shell. Fluorescent staining revealed developmental delay of the shell field at low pH, and abnormal development thereof was correlated with hinge defects in D-veligers. This study shows, for the first time, that ocean acidification affects larval soft-tissue development, independent from calcification. Multiple developmental processes additively underpin the teratogenic effect of ocean acidification on bivalve larvae. These results explain why trochophores are the most sensitive life-history stage in marine bivalves and suggest that short-term variability in carbonate chemistry can impact early larval development.

## 1. Introduction

Coastal marine ecosystems experience dynamic spatio-temporal variability in seawater inorganic carbonate chemistry [1]. Short-term variability occurs on top of baseline changes associated with ocean acidification. Ocean acidification causes a decrease in mean seawater pH and aragonite saturation state (*Ω*_a_) via absorption of anthropogenic carbon dioxide (CO_2_) emissions [2]. As ocean acidification progresses, the acidity of naturally occurring low pH events is enhanced [3]. How the interplay of acidification and coastal pH variability will impact marine organisms requires understanding what aspect of pH variability influences biological processes that occur over similar time frames [4,5].

Fluctuations in coastal carbonate chemistry often occur on a diel period. Diel fluctuations result from daytime photosynthetic removal of CO_2_ by phytoplankton and benthic photoautotrophs followed by night-time respiration of the whole community [6]. Depending on habitat characteristics, seagrasses, macroalgae and kelp forests can induce diel pH fluctuations that span a few tenths to a full pH unit [5–8]. These short-term pH fluctuations are of similar magnitude as anthropogenic ocean acidification (−0.4 units pH by end of the century) [9], which is known to affect a myriad of biological processes [10]. In addition to baseline changes in carbonate chemistry, ocean acidification is expected to increase the magnitude of diel fluctuations [5].

The biological impact of fluctuating carbonate chemistry in the context of ocean acidification is understudied [11]. A few recent studies that address this issue show non-generalizable responses across calcifying taxa and species [11,12]. For molluscs, one the most studied taxa, growth appears largely indifferent to variability [13–16] (but see [17]) despite the fact that exposure to fluctuating conditions may be energetically costly [18]. Understanding impacts of variability requires knowing which parameter of carbonate chemistry drives a biological response and the reaction norm thereof. Given recent advances in these criteria for molluscs, here we focus on the early development of bivalve larvae.

The early development of bivalves is completed within 2–3 days after fertilization and is extremely sensitive to carbonate chemistry [19–22]. Specifically, abnormal development and reduced growth of D-veliger larvae occur under conditions of low *Ω*_a_ or low substrate inhibitor ratio $\left(\mathrm{SIR}=[\mathrm{HC}O_{3}^{-}]/[H^{+}]\right)$ [23,24], which are tightly coupled in manipulations using CO_2_. Previous work suggests that the first 24 h of development are insensitive to CO_2_ acidification and exposure thereafter drives abnormal development [21]. Subsequent shell growth is highly dependent on *Ω*_a_ or SIR [23,24], due to the fact that larvae have limited control over carbonate chemistry at the site of calcification [25]. These findings suggest that early development of bivalves is comprised of process-specific sensitivities, and short-term fluctuations in carbonate chemistry typical of shallow coastal waters may be important to larval development. Despite its environmental relevance, only a few studies have investigated the impact of variable carbonate chemistry on early development of marine bivalves [13,14], with inconsistent outcomes across species [17]. For example, Frieder *et al.* [17] found that semi-diel variability enhanced larval growth in *Mytilus galloprovincialis*, but not in *M. californianus*. As the aforementioned studies do not control for timing of fluctuations, it remains unclear how fluctuating carbonate chemistry affects specific developmental processes in bivalve larvae.

The aim of this study was to identify what aspects of diel fluctuations in carbonate chemistry (e.g. timing, magnitude) are important to the early growth and development of the mussel *M. galloprovincialis*. Global aquaculture of *M. galloprovincialis* largely depends on natural recruitment for seed supply [26], so natural variability in seawater chemistry in the context of ocean acidification is highly relevant to the persistence of this industry. The first experiment (Exp. 1) tests the hypothesis that the magnitude of variability influences larval growth and development. Results from Exp. 1 informed the design of Exp. 2 and Exp. 3, which test the hypothesis that development is influenced by the timing of variable exposures. A fourth experiment (Exp. 4) was conducted to specifically test the hypothesis that CO_2_-acidified seawater alters development of the trochophore shell field. For two of the four experiments, larvae from unique parental crosses were cultured in isolation to explore the biological variation of the response across parental pairs (Exp. 2 and Exp. 4).

## 2. Material and methods

For reading simplicity and based on methods of manipulation, we describe treatments in terms of pH, whereby low pH represents the full suite of chemical changes brought on by CO_2_-acidification under stable temperature and salinity (electronic supplementary material, table S1). Low pH treatments were chosen to reach levels of aragonite undersaturation known to induce abnormal development and reduced larval growth [24].

### (a) Experimental design

**Figure 1.**
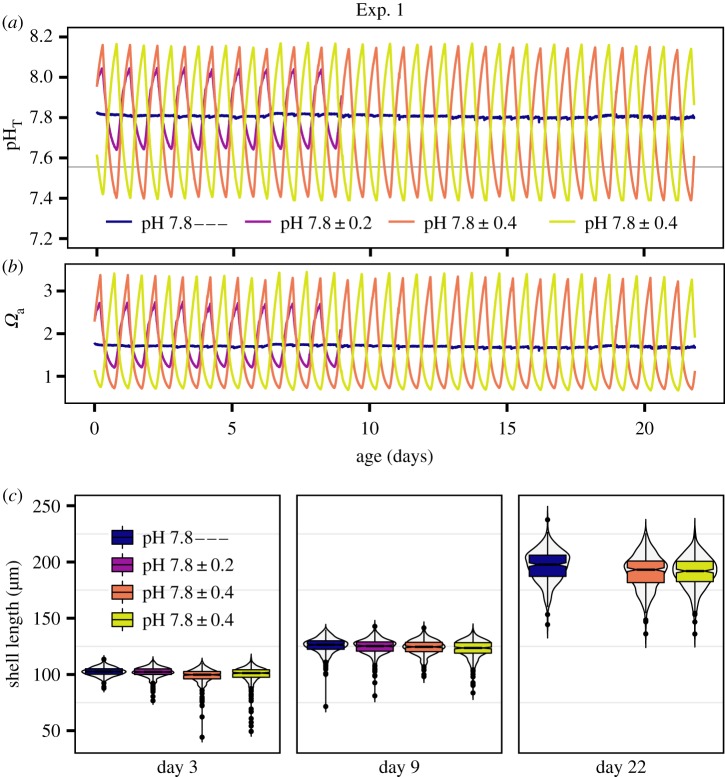
D-veliger shell size correlates to mean exposures: Exp. 1. (*a*) pH time series of Exp. 1, grey references *Ω*_a_ = 1, hpf is hours post-fertilization. (*b*) *Ω*_a_ time series of Exp. 1. (*c*) Shell size of larvae in Exp. 1 on days 3, 9 and 22. Box plots denote median, median confidence interval (notch width), quartiles and outliers (dots). Violin plots show the size distribution of the observations (*n* ≈ 300).

In the context of ocean acidification in dynamic coastal zones, a range of variable pH treatments was used (pH_T_ 7.4–8.1). Exp. 1 assessed the impact of stable compared to variable pH treatments with the same mean pH (figure 1; electronic supplementary material, table S1). Four treatments were set up: control treatment of stable pH_T_ 7.8 (pH 7.8—), and three variability treatments with a mean pH_T_ 7.8 and a diel range of either 0.4 (pH 7.8 ± 0.2), 0.8 (pH 7.8 ± 0.4), or 0.8 offset by 12 h (pH 7.8 ± 0.4), in order to control for the timing of variability exposures. Embryos from three unique families, using three males and nine females (1 male × 3 females, replicated three times with unique individuals), were cultured separately in the four treatments (12 cultures total, *N* = 3 biological replicates). Larvae were sampled on day 3 (size and morphology), 9 (size) and 22 (size). Treatment pH 7.8 ± 0.2 was discontinued after day 9 for technical reasons.

**Figure 2.**
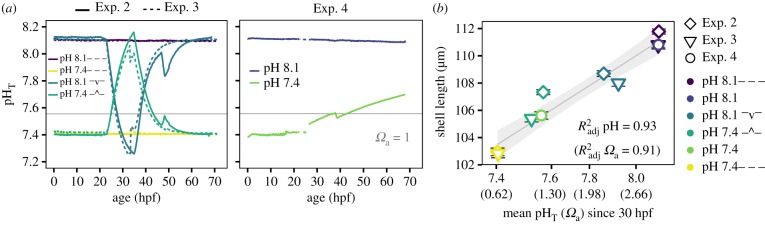
D-veliger shell size correlates to mean exposures: Exp. 2–4. (*a*) pH time series of Exp. 2–4, grey references *Ω*_a_ = 1, hpf is hours post-fertilization. (*b*) Mean shell size (*n* ≈ 300, ±s.e.) of D-veligers from Exp. 2–4 (symbols) on day 3 plotted against the treatment-respective mean pH exposure since the start of calcification (30 hpf) to sampling time (68–72 hpf). Mechanistically, as calcification is driven by *Ω*_a_ (or SIR) and not pH, the corresponding *Ω*_a_ data is shown in parentheses (*x*-axis, $R_{\mathrm{adj}}^{2}$). Colours indicate pH treatments in (*a*). Linear regression for pH is in grey (±s.e.).

Variable pH treatments in Exp. 2 and Exp. 3 (figure 2*a*; electronic supplementary material, table S1) were designed to assess the effect of pH exposure at the start of shell morphogenesis, using a single diel pH fluctuation down from pH_T_ 8.1 (pH 8.1**^–^**v**^–^**) or up from pH_T_ 7.4 (pH 7.4**–^–**) centred around the start of calcification, approximately 30 hours post-fertilization (hpf). Stable pH_T_ exposures were used as controls (pH 8.1— and pH 7.4—) and larvae were sampled on day 3 (size and morphology). In Exp. 2, embryos from three unique pairs were cultured separately (12 cultures in total, *n* = 3 biological replicates per treatment). In Exp. 3, embryos from nine unique pairs were pooled and then distributed across three replicate cultures (total of 12 cultures, *n* = 3 technical replicates). In Exp. 4 (figure 2*a*), embryos from five unique pairs were cultured separately in pH_T_ 8.1 and pH_T_ 7.4 (10 cultures in total, *n* = 5 biological replicates per treatment) and sampled at two time points: 35 hpf (fluorescent staining) and 68 hpf on day 3 (size, morphology, scanning electron microscopy [SEM]).

### (b) Larval cultures

Gravid *M. galloprovincialis* were collected from two sites during local spawning seasons. The first group was collected from a dock in Thau Lagoon (43.415°N, 3.688°E), a shallow semi-enclosed bay in Sète, France on 11 October 2016 (Exp. 1). The second group was collected from shallow buoy lines in the Bay of Villefranche-sur-Mer (43.682°N, 7.319°E), France on 9 February 2017 (Exp. 2–4). During the spawning season, pH variability in Thau Lagoon ranged from pH_T_ 7.80 to 8.10 (L.K., 2016, unpublished data) and from pH_T_ 8.10 to 8.15 in the Bay of Villefranche [27]. Adult mussels were kept in a single temperature-controlled flow-through sea table (approx. 16°C) and fed three times per week a mixture of Instant Algae (Reed Mariculture, Iso 1800 and Shellfish Diet 1800) until spawning. Experiments were conducted from January through March 2017 (see electronic supplementary material). Spawning was induced by cleaning the mussels of epibionts and warming seawater to 28°C. Sperm was kept on ice and eggs were kept at approximately 16°C until fertilization. Test fertilizations were performed to ensure gamete compatibility prior to batch fertilization. Fertilization was performed simultaneously across biological replicates, except for Exp. 4, which required 30 min staggered fertilization by pair to facilitate sampling at precisely 35 hpf. Successful fertilization was determined by greater than 90% presence of polar bodies, after which embryos were transferred to culture vessels at a density of 14 embryos ml^−1^. All experiments were run at 14.3°C (near February habitat conditions [27]) to maintain equal developmental rates. In Exp. 1, larvae were fed 1 × 10^8^ live cells of *Tisochrysis lutea* (CCAP 92714) once or twice per day after day 3.

### (c) Experimental system and seawater chemistry

Larvae were cultured in a temperature-controlled pH variability system described in Kapsenberg *et al.* [28]. Four header tanks (35 l) were supplied with seawater pumped from 5 m depth in the Bay Villefranche, filtered to 0.35 µm and UV-sterilized (FSW). Header tank pH was manipulated via the addition of pure CO_2_ gas using a glass pH electrode feedback system (IKS Aquastar) and constant aeration with CO_2_-free air. Treatment water was pumped from header tanks to culture buckets (*n* = 3 per header tank, see electronic supplementary material for control of header tank effects) using irrigation drippers (2 l h^−1^). Variable pH treatments switched header tank pH every 12 h, resulting in smooth pH oscillations in cultures as documented by Honeywell Durafet III pH sensors (for performance quality see electronic supplementary material). Exp. 4 consisted of static cultures (sourced from two independent header tanks of pH_T_ 8.1 and pH_T_ 7.4) due to the use of calcein dye. Water samples were collected from each treatment header tank for salinity (Mettler Toledo SevenEasy Conductivity) and total alkalinity (*A*_T_) every 2–3 days for Exp. 1, every 2 days for Exp. 2, every day during Exp. 3, and once at the start of Exp. 4. Samples for *A*_T_ were run in duplicate using open cell titration (Metrohm Titrando 888) [29]. Accuracy of *A*_T_ measurements ranged between −7 and +5 µmol kg^−1^ as compared to certified reference material (A. Dickson, Scripps Institution of Oceanography). Precision of *A*_T_ measurements was 2 µmol kg^−1^ (mean standard deviation of all duplicate measurements, *n* = 68). *Ω*_a_ and *p*CO_2_ were calculated from pH_T_ using mean temperature, and header tank salinity and *A*_T_ per treatment, per experiment (electronic supplementary material, table S1). Carbonate system calculations were performed in RStudio (version 1.0.143) using the seacarb R package [30], with constants *K*_1_ and *K*_2_, *K*_f_, and *K*_s_ from Lueker *et al.* [31], Perez *et al.* [32] and Dickson [33], respectively.

### (d) Shell size, morphology and SEM

Shell size was determined as the maximum shell length parallel to the hinge, using bright field microscopy and ImageJ software (*n* ≥ 100 culture^−1^). On day 3, larval morphology was scored (*n* ≥ 100 culture^−1^) according to His *et al.* [34]: normal (D-shaped shell with a straight hinge), trochophore (undeveloped larvae, often shell-less), abnormal hinge (concave D-shaped shell), protruding mantle (D-shaped shell with protruding tissue or velum), and combined abnormal hinge and protruding mantle (D-shaped shell with both abnormalities). For SEM imaging, D-veligers were preserved in 100% EtOH. Prior to imaging (JEOl 6010LV), shells were cleaned (rinses of 2 min tap water, 5 min R.O. H_2_O with 1% bleach, 2 min tap water, 5 s ddH_2_O, temporary storage in 100% EtOH), dried at 42°C, and sputter coated with gold.

### (e) Fluorescent staining

To visualize the first CaCO_3_ precipitation, calcein (0.001 M, final concentration) was added to static cultures of Pair 2 and 4 at 25 hpf in Exp. 4 [35]. Pilot experiments using calcein dye showed that the first calcification occurred during the early trochophore stage at 30 hpf in 14.3°C. Immediately after calcein addition, motorized paddles were turned on in all culture vessels, resulting in slight CO_2_ off-gassing in pH_T_ 7.4. Calcein culture pH was checked daily using a glass pH electrode which was compared against a calibrated Durafet (addition of calcein caused a 0.03 unit pH_T_ increase in the high pH treatment and 0.10 unit pH_T_ decrease in the low pH treatment).

To visualize the organic matrix of trochophores in Exp. 4, larvae from all pairs were sampled and stained live with calcofluor (Calcofluor White M2R, #F3543 from Sigma-Aldrich) at 35 hpf. Calcofluor is a fluorochrome that binds to chitin (and cellulose) and has been used to study chitin in adult abalone [36]. Its precision for identifying chitin from other matrix molecules has been debated [37] and the results presented here are restricted to assessing the shape and extent of the organic matrix in general. Concentrated larvae were stained by calcofluor in FSW for 5 min (final exposure of 1 : 50 000 w/v by diluting a stock solution of 1 : 100 w/v in DMSO, stored at −20°C), washed three times with FSW, fixed with a drop of 4% paraformaldehyde, and immediately imaged on a confocal microscope (Leica SP8, electronic supplementary material). Images were 3D rendered and composite images were rotated for each larva to measure the area of one valve stained by calcein and calcofluor via manual drawing using ImageJ software (*n* = 13–42 culture^−1^).

### (f) Statistical analyses

Data analysis was performed in RStudio (v. 1.0.143) [38]. Residuals of larval shell size data were not normally distributed, violating assumptions required for ANOVAs. For Exp. 1, a two-factor permutation analysis of variance was used with 1000 permutations in RVAideMemoire R package [39]. Treatment and age were fixed factors (interaction was not significant and removed from the final model) and family was used as a blocking factor. Size data from the first 100 larval measurements were used and data from treatment pH 7.8 ± 0.2, stopped after day 9, was excluded. For Exp. 2–4, a linear regression was used to assess the impact of mean pH exposure on mean larval size during the shell growing period (30–68 hpf). Exp. 2–4 had different biological design (isolating versus pooling larvae from unique parental pairs), so a linear regression was performed on overall mean larval size (*n* ≈ 300 treatment^−1^ experiment^−1^). Cultures with calcein dye in Exp. 4 were excluded as these cultures did not contain a Durafet by which to calculate the mean pH exposure. For Exp. 1 and Exp. 2 (family and pair replication), proportions of larvae with normal development was analysed using a generalized linear mixed effects model with treatment as a fixed effect and family or pair as a random effect, using the lmer R package [40]. Significance of the fixed effect was tested against the null model using a likelihood ratio test. For Exp. 3 (bucket replication), treatment effect was assessed using a one-way ANOVA. Analyses were repeated for Exp. 2 and Exp. 3 for proportion of larvae with an abnormal hinge (irrespective of a protruding mantle). All model residuals exhibited a normal distribution (Shapiro–Wilk normality test) and equal variance (Levene's test). Least-squares means were used for *post hoc* pairwise contrasts of treatment effects for Exp. 2 and Exp. 3, using a Bonferroni correction for six comparisons in the lsmeans R package [41].

## 3. Results

### (a) Shell size responds to mean conditions

The impact of variable carbonate chemistry on shell growth was analysed by comparing shell length of larvae reared under treatments of either constant pH_T_ 7.8 or mean pH_T_ 7.8 with a total diel range of 0.4 or 0.8 units pH_T_ in Exp. 1 (figure 1). Shell length was not significantly affected by a 0.8 unit pH_T_ diel variation over the course of a three-week exposure (figure 1*c*; treatment effect: *F*_1,2_ 3.33, *p =* 0.093; shell size increased over time, *F*_1,2_ 1712.68, *p* < 0.001), which suggests that shell length is a function of mean exposures. This was empirically tested and verified in Exp. 2–4, using a combination of stable and variable treatments spanning a range of mean exposures (figure 2). Shell length was significantly correlated to mean exposures during the shell growing period, 30 hpf until the sampling time on day 3 (for pH: *F*_1,8_ 119.6, *p* < 0.0001; for *Ω*_a_: *F*_1,8_ 92.57, *p* < 0.0001). Mean pH_T_ (*Ω*_a_) explained 93% (91%) of the variance in average shell size of larvae across treatments, respectively (figure 2*b*). This correlation was independent of developmental effects (abnormal larvae are smaller than normal D-veligers in low pH treatments; electronic supplementary material, figure S1 and table S2) as the correlation was maintained for larvae in the top 10th percentile size class (for *Ω*_a_, *F*_1,8_ 32.27, *p* < 0.001, $R_{\mathrm{adj}}^{2}=0.78$).

### (b) Larval development depends on timing of exposure to acidified seawater

**Figure 3.**
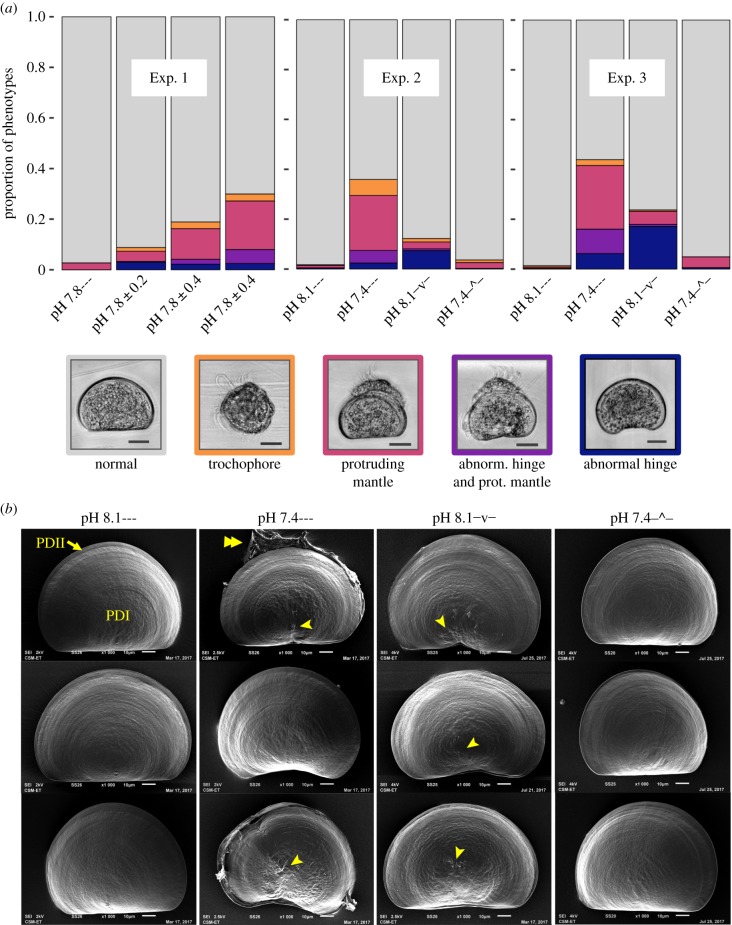
Variable pH alters development of mussel larvae. (*a*) Proportion of larval phenotypes on day 3 in Exp. 1, Exp. 2 and Exp. 3. Treatment labels match those in figures 1*a* and 2*a*. Colour coding follows the border colour of representative phenotype images (scale bar = 30 µm). Per experiment, proportions of specific phenotypes were calculated from summed observations across three cultures per treatment (*n* ≈ 300; see text for design of biological replication of cultures). (*b*) SEM images of D-veliger shells observed on day 3 in Exp. 3 (scale bar = 10 µm, brightness and contrast was adjusted per image). Examples of three larvae were chosen to highlight phenotypes associated with treatment effects. Prodissoconch I (PDI) and II (PDII) is indicated for a larva from pH 8.1— (top). Examples from pH 7.4— and pH 8.1^–^v^–^ show larvae with abnormal hinges and scarring at the centre of PDI (arrowhead). Larva with a protruding mantle is indicated by a double arrowhead.

In Exp. 1, abnormal development increased with the magnitude of the diel pH range (figure 3*a*; *p*-value < 0.007 for all pairwise comparisons, electronic supplementary material, table S3). The increase in abnormal development across treatments with diel cycles offset by 12 h (pH 7.8 ± 0.4 versus pH 7.8 ± 0.4; *p* = 0.0013) suggests that the timing of low pH conditions was an important factor for developmental outcomes. This was empirically tested and verified in Exp. 2 and Exp. 3 (electronic supplementary material, tables S4 and S5). In both Exp. 2 and Exp. 3, the most striking result was that ≥95% of larvae developed normally in both pH 8.1— and pH 7.4**–^–** (figure 3*a*), whereas larvae in pH 8.1**^–^**v**^–^** exhibited 76–88% normal development, mostly due to the presence of abnormal hinge phenotypes. In pH 7.4—, only 56–64% of larvae exhibited normal development due to frequent observations of larvae with protruding mantles and abnormal hinges. Larvae with both an abnormal hinge and protruding mantle were most prevalent in pH 7.4— (5.1% in Exp. 2, and 9.8% in Exp. 3), wherein 60–70% of larvae with a hinge abnormality also exhibited a protruding mantle. The combination of an abnormal hinge and a protruding mantle was observed in less than 1% of larvae in pH 8.1**^–^**v**^–^** (due to few protruding mantles), and never in pH 8.1— and pH 7.4–^– (due to absence of abnormal hinges). The temporal partitioning of unfavourable seawater chemistry exposure and associated abnormal phenotypes suggests that abnormalities in the hinge and mantle are additive. This additive effect was also apparent in the shell size of these phenotypes (electronic supplementary material, figure S1): larvae with an abnormal hinge, protruding mantle or both are 1, 4 and 6% smaller, respectively, than normal D-veligers from the same low pH treatment (electronic supplementary material, table S2).

### (c) Abnormal development of the shell field is correlated to hinge abnormalities

In both Exp. 2 and Exp. 3, the proportion of larvae with an abnormal hinge (irrespective of a protruding mantle) was significantly greater in treatments that experienced low pH conditions around 30 hpf (7–8% in Exp. 2, 16–18% in Exp. 3, in pH 7.4— and 8.1**^–^**v**^–^**) compared to those that experienced high pH at this time (less than 1% in pH 8.1— and pH_T_ 7.4**–^–**; pairwise comparisons *p*-values ≤0.004; electronic supplementary material, tables S6 and S7) and equal between treatments which experienced the same pH at that time (*p*-values = 1.0; electronic supplementary material, tables S6 and S7). Therefore, hinge abnormalities were induced by exposure to unfavourable low pH conditions around 30 hpf (27–35 hpf), independent of prior or later exposures. Larvae exposed to unfavourable carbonate chemistry during this period frequently exhibited irregular texture and scarring in the centre of the first larval shell (PDI, prodissoconch I) (e.g. figure 3*b*; in pH 7.4— and pH 8.1**^–^**v**^–^** in Exp. 3). In contrast, D-veliger shells of larvae reared in pH 8.1— and pH 7.4**–^–** were indistinguishable (figure 3*b*).

**Figure 4.**
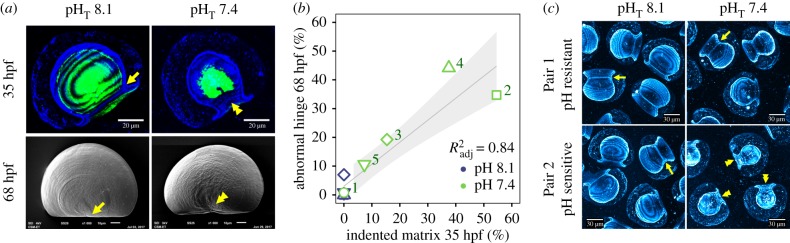
Relationship between organic matrix morphology and D-veliger shell phenotype in Exp. 4. (*a*) Images of larvae in pH_T_ 8.1 (straight hinge, arrow) and pH_T_ 7.4 (indented matrix and hinge, double arrowhead). Composite confocal images show larvae at 35 hours post-fertilization (hpf; organic matrix stained in blue, CaCO_3_ stained in green, scale bar = 20 µm) and SEM show larvae at 68 hpf (scale bar = 10 µm). Larvae are of unique individuals from Pair 2 and were selected to best visualize the correlation shown in (*b*). (*b*) Percentage of larvae with an indented organic matrix at 35 hpf (*n* = 13–42) correlated with percentage of D-veliger larvae exhibiting an abnormal hinge at 68 hpf (*n* = 109–155), across five unique male–female pairs (symbol, numbered for pH_T_ 7.4). Linear regression line is grey (±s.e.). (*c*) Trochophore larvae (Pair 1 and Pair 2) reared in pH_T_ 8.1 and pH_T_ 7.4 and stained with calcofluor (cyan) at 35 hpf. Colour was adjusted equally across images for clarity of the hinge line (scale bar = 30 µm). Arrows denote normal organic matrices and hinge lines (arrow) and indented matrix (double arrowhead). Ring-like pattern in staining is an artefact of confocal imaging larvae through multiple slices.

The developmental process underpinning the abnormal shell development was investigated in Exp. 4. In pH_T_ 7.4 at 35 hpf, the organic matrix of some larvae exhibited an indentation along the hinge line (figure 4*a*). In contrast, in pH_T_ 8.1, this chitinous isthmus was straight and well developed. The proportion of larvae with an indented matrix at 35 hpf was highly correlated to the proportion of D-veligers with an abnormal hinge at 68 hpf (figure 4*b*; *F*_1,8_ = 47.89, *p* = 0.0001, $R_{\mathrm{adj}}^{2}=0.84$). Larvae from Pair 1 exhibited greater than 99% normal development in pH_T_ 7.4 at both trochophore (35 hpf) and D-veliger stage (68 hpf), with normal flat shell matrices in both pH_T_ 8.1 and pH_T_ 7.4 at 35 hpf (figure 4*c*). In contrast, 55% of larvae from Pair 2 exhibited matrix indentations in pH_T_ 7.4 (*n* = 12, of 22 larvae), and 35% of D-veligers exhibited hinge abnormalities by day 3 (*n* = 42, of 121 larvae). While some variation in the correlation between phenotypes at 35 and 68 hpf may be related to sample size, these data, along with results from Exp. 2 and 3, provide strong evidence that matrix indentations persists throughout development and cause hinge abnormalities in D-veligers.

Matrix indentations occurred in the presence and absence of CaCO_3_ (electronic supplementary material, figure S2), suggesting that abnormal shell field development is a phenotype that is fixed prior to calcification. At 35 hpf, only 87% of pH_T_ 7.4 larvae had started calcification (*n* = 40 out of 46). Shell abnormalities in pH_T_ 7.4 were visible as keyhole-shaped indentations near the hinge, indicating no calcification in that region by 35 hpf (figure 4*a*). In pH_T_ 8.1, this abnormality was not observed and all larvae exhibited substantial shell growth (*n* = 58). Both the organic matrix and larval shell were larger for larvae reared in pH_T_ 8.1 compared with those in pH_T_ 7.4 at 35 hpf (electronic supplementary material, figure S3), regardless of the normal or abnormal developmental trajectory of the larval cohort (visually apparent in figure 4*a*,*c*).

### (d) Protruding mantle indicates sensitivity of the PDI-II transition

The protruding mantle phenotype occurred during the early D-veliger stage around 40 hpf. In Exp. 2 and 3, larvae with protruding mantles (irrespective of an abnormal hinge) were associated with low pH exposure, with approximately 30% observed in pH 7.4— and ≤1% in pH 8.1— (figure 3*a*). In contrast, treatments pH 7.4–^– and pH 8.1^–^v^–^ induced a low occurrence of protruding mantle (respectively, 2.3 versus 3.5% in Exp. 2; 4.3 versus 6.1% in Exp. 3) indicating that this phenotype was induced by low pH at a time when pH in both treatments were similar. There are only two periods where this occurs, near 27 and 40 hpf. The most relevant time-point is 40 hpf, as embryos are resistant to CO_2_ acidification during the first day of development [21], and developmental processes around 40 hpf relate to the shell–mantle interface at the transition from the first (PDI) to the second larval shell (PDII).

## 4. Discussion

The aim of this study was to identify which aspect of mussel larval development is influenced by temporal variability of carbonate chemistry. By using a range of variability treatments, we show that shell growth responds to mean exposures while development depends on time-sensitive exposures (Exp. 1–4), and these processes additively contribute to overall D-veliger phenotypes. Based on timing of unfavourable conditions, sensitive developmental processes were identified and linked to specific abnormal phenotypes (Exp. 2–3), with mechanistic evidence at the tissue layer (Exp. 4). Electronic supplementary material, figure S4 details the developmental timeline and windows of sensitivity.

### (a) Mussel larval growth and development

Larval shell growth was driven by mean exposures regardless of variability regimes (figures 1*c* and 2*b*). This reflects the instantaneous nature of *Ω*_a_-dependent precipitation kinetics in bivalve larvae [42], whereby the benefit of high *Ω*_a_ is neutralized by the negative effect of low *Ω*_a_. This has also been observed in other mollusc larvae, including abalone, hard clams, oysters and scallops [13–15], although oddly not for *M. galloprovincialis* from California [17]. Frieder *et al.* [17] found that *M. galloprovincialis* larvae increased growth in low pH treatments when semi-diurnal fluctuations were included. Culture temperatures and larval growth rates in control treatments in their study and ours are comparable (approx. 125 µm; day 8 in *Ω*_a_ 1.9 at 15.7°C versus day 9 in *Ω*_a_ 1.7 at 14.4°C in our study). As Frieder *et al.* [17] conducted a single experiment with few replicate measurements, further experiments will be necessary to identify the source of these contrasting observations.

In terms of development, we identified two processes that, when they occur during conditions of low pH (low *Ω*_a_ and SIR), give rise to abnormal D-veligers: (i) formation of the shell field prior to calcification around 30 hpf, and (ii) velum retraction around 40 hpf prior to the transition from PDI to PDII.

Exposure to low pH conditions around 30 hpf produced D-veligers with an abnormal hinge (figure 3, Exp. 2 and 3). The major developmental process occurring at this time is the formation of the shell field during the mid-trochophore stage (electronic supplementary material, figure S4). This process is initiated in the early trochophore stage by the invagination of a group of ectoderm cells, which create a pore. This brings together a rosette of outer surface cells that secrete what appears to eventually become the periostracum [43]. The pore closes and deeper invaginated cells then evaginate back to the surface epithelium where, under control conditions, they create a flat region that expands via mitotic division, under the expanding periostracum [43–45]. During this process, shell field cells exude a chitin-based organic matrix wherein calcification takes place [43,46,47]. In low pH treatments, the organic matrix often exhibited a central indentation at the site of the shell field invagination (figure 4). At 35 hpf, the lack of calcification in this area and scarring in the centre of PDI on D-veligers suggests a treatment effect on cells associated with the shell field evagination (figure 3*b*). Larvae in pH 7.4**–^–** calcified smooth shells, despite being in unfavourable carbonate chemistry from approximately 40 hpf onward. This indicates that the effect of unfavourable carbonate chemistry at the start of calcification (approx. 30 hpf), which generated abnormal shell texture, is different from the effect of low *Ω*_a_ that drives the rate of linear, but smooth, extension of the shells after this period [24]. Similar distortions in PDI have previously been observed in oyster larvae of *Ostrea edulis* and linked to the process of the shell field evagination, although this was not in the context of environmental conditions [48]. Hinge abnormalities may result from abnormal or incomplete restructuring of the ectoderm during development of the shell field, probably prior to calcification. The resulting abnormal trochophore body shape alters the calcification blueprint, whereby the shell simply takes on the shape of the cellular landscape over which the organic matrix is exuded, thereby producing D-veligers with an indented hinge.

Within low pH treatments, the abnormal hinge phenotype is only 1% smaller than normal D-veligers (electronic supplementary material, figure S1). In Exp. 1, abnormal hinge larvae were still present on day 9 (L.K. 2017, personal observation). By day 22, however, curvature of the shell masked the angle of the shell hinge, so there is no evidence to infer the effect of the abnormal hinge phenotype on larval fitness.

Regardless of normal or abnormal shell field development, the organic matrix and calcified area at 35 hpf was consistently smaller in low pH treatments, which we interpret as developmental delay. Developmental delay likely occurs from exposures during the early trochophore stage (electronic supplementary material, figure S4). The energetic cost of building the protein-rich organic matrix is much greater than the cost of external calcification [49,50]. In oyster larvae, low pH causes a decrease in the protein deposition efficiency [51]. Developmental delay of the shell field may thus stem from protein production issues associated with building the organic matrix. Consequently, this could also delay the onset of calcification.

Exposure to low pH conditions near 40 hpf was linked to increased frequency of D-veligers with protruding mantle tissue or velum (irrespective of an abnormal hinge). At this time, the extension of the calcified PDI shell has caught up with the leading edge of the expanding organic matrix, which then both fully cover the larval body (L.K. 2017, personal observation). Around 47 hpf, larvae gain the ability to retract their velum and close their shell (a behavioural response to ethanol exposure; L.K. 2016, personal observation). Shell closure marks the transition from PDI growth to concentric growth lines distinctive of PDII (electronic supplementary material, figure S5) [48]. Protruding tissue is evidence of either abnormal tissue development or inability to retract the velum. It is likely that these larvae cannot progress in development to PDII growth. Such a developmental arrest is evident in shell size. D-veligers with protruding tissue tend to be 4–6% smaller than normal D-veligers (electronic supplementary material, figure S1). Previous experiments showed that protruding mantle phenotypes of *M. galloprovincialis* lacked PDII growth, even after a 5-day exposure to *Ω*_a_ 0.49 [21], which suggests that this phenotype is terminal.

From this study, it is clear that CO_2_-acidified seawater impacts development of the larval body in multiple ways, independent from impacts on calcification (electronic supplementary material, figure S4). This study raises the question as to how seawater CO_2_-acidification disrupts tissue development in mussel larvae and highlights the need to identify mechanisms of ocean acidification impacts at a cellular level.

### (b) Environmental and global change context

Our results suggest that developmental success of mussel larvae will be unpredictable in habitats with high variability in carbonate chemistry. Cues that determine the moment of a spawning event in the field are not well understood. The mussel population in Villefranche appears to spawn out during storms, and mussels have spawned in a bucket on the boat, which suggests that the final trigger may be physical and unrelated to time of day. Temperature-dependent developmental rates could also influence when a given larval cohort will be sensitive to carbonate chemistry. As shell growth depends on mean conditions, areas with high mean *Ω*_a_, regardless of variability regimes, may significantly benefit normally-developed D-veliger larvae, if larvae are retained in this body of water throughout their pelagic phase.

Normal larval development in pH_T_ 7.4 ranged from 30% to 99% across unique parental pairs (electronic supplementary material, figures S6 and S7). Such biological variation is common among bivalves and may facilitate adaptation to global change [52,53]. Consequently, as ocean acidification progresses, dormant genotypes previously unselected for in natural populations may be favoured via new natural selection pressures operating on early development [54]. Although we did not test for this, evidence for adaptation can be found via population comparisons between sites with different pH regimes [55]. Overall, future recruitment of *M. galloprovincialis* is likely to increase in variance as emerging factors such as local carbonate chemistry variability, timing of spawning, parental effects, and other co-occurring global change stressors gain importance in determining successful development of a larval cohort.

## 5. Conclusion

By controlling the timing of low pH events, this study partitions the effect of ocean acidification on mussel larval growth from that on development. Larval growth is a function of mean exposures. This extends previous research on the *Ω*_a_-dependency of calcification in bivalve larvae [23,24] by demonstrating that calcification responds instantaneously to changes in seawater chemistry. Independent from calcification, abnormal development was driven by sensitivity to low pH conditions during specific soft-tissue developmental processes: (i) formation of the shell field and (ii) transition from PDI to PDII. This is the first study documenting ocean acidification sensitivity in soft tissues of bivalve larvae and how formation thereof determines D-veliger morphology. These additive and short-lived processes explain why trochophores are the most sensitive life-history stage in marine bivalves.

## Supplementary Material

Supporting Information

## Data accessibility

Data are archived on BCO-DMO, https://www.bco-dmo.org/project/720349.
