# Supplementary material for: Ocean pH fluctuations affect mussel larvae at key developmental transitions
Source: Proc Biol Sci. 2018 Dec 19;285(1893):20182381. doi: 10.1098/rspb.2018.2381 (PMC6304040; doi:10.1098/rspb.2018.2381)
Supplement: Supporting Information [file rspb20182381supp1.pdf]

Supporting Information

Ocean pH fluctuations affect mussel larvae at key developmental transitions

L. Kapsenberg, A. Miglioli, M. C. Bitter, E. Tambutté, R. Dumollard, J.-P. Gattuso

This PDF file includes:

- Supplementary methods
- Figs. S1 to S7
- Tables S1 to S7
- References for SI reference citations

Supporting Methods

Experiment dates. Exp. 1 was started on 17 January 2017, using mussels collected in Sète, France, on 11 October 2016. Exp. 2, 3, and 4 were started on 14 February, 21 February, and 6 March 2017, respectively, using mussels collected from the Bay of Villefranche-sur-Mer, France, on 9 February 2017.

Header tank effects. In Exp. 1-3, culture replicates received water from the same header tank. Exp. 1 is most affected by this as this was the longest experiment. However, we did not observe visual differences in header tanks in terms of biofouling, and results from Exp. 1 are consistent with the results from Exp. 2-4. We purposely repeated experiments (Exp. 2, Exp. 3) to address potential header tank affect (none observed), randomly switched treatments assigned to header tanks between Exp. 2-4, and used two independent header tanks per treatment in Exp. 4.

Size of phenotypes at low pH (Fig. S1, Table S2). Size class of each abnormal D-veliger phenotype in low pH was assessed using data from experiments which isolated larval cultures by parental pair: Exp. 2 (pH_T 7.4---) and Exp. 4 (pH_T 7.4). As this was a *post hoc* analysis, size measurements were non-random because additional photos were used to increase sample size of phenotypes with low frequencies. Phenotype categories with less than 5 final counts per culture were excluded. Data from Exp. 3 Pair 3 was excluded due to uncertainty in scoring protruding mantle phenotypes due to tissue disintegration (this did not interfere with other presented analyses and conclusions). Size class of abnormal phenotypes, relative to normal D-veligers, was assessed used a linear quantile mixed model (R package lqmm [1, 2]). These models do not have assumptions of normality or equal sample size and allow for random effects. D-veliger phenotype was considered a fixed effect while pair (N=7) was considered a random effect on the intercept. Quantiles of abnormal phenotypes were compared to those of normal D-veligers (block-bootstrap, N=50).

Durafet calibration and performance. Prior to each experiment, Durafets were calibrated with spectrophotometric pH measurement using purified m-cresol purple (R. H. Byrne, University of South Florida) and calibration was checked at the end of each experiment [3]. Agreement among calibrated Durafets across a pH range of pH_T 6 to 8 was ± 0.005 units pH_T or better, throughout the experimental period. The accuracy of the pH time series is better than ± 0.01 units pH_T; mainly limited by the ± 0.008 unit uncertainty of the spectrophotometric calibration [3]. For Exp. 1, post-experiment Durafet pH offset from spectrophotometric pH measurements ranged from 0.003 to 0.014 (potentially due to biofouling by live phytoplankton used to feed larvae). For Exp. 2-4, post-experiment Durafet pH offset from spectrophotometric pH measurements was on average -0.001 ± 0.004 units (N=12), including a -0.009 unit offset in Exp. 2 (pH_T 7.4-^-) and +0.009 unit offset in Exp. 3 (pH_T 7.4 for Pair 4 and 5 only). Durafet data was logged on a 5 min frequency.

Confocal imaging. Calcofluor was excited with the 408 blue diode laser and emitted fluorescence was collected between 450 and 490 nm. Calcein was excited with the 488 nm laser line and fluorescence collected between 520 and 560 nm. A z-stack of 20 images encompassing the whole volume of the larvae was imaged for each larva.

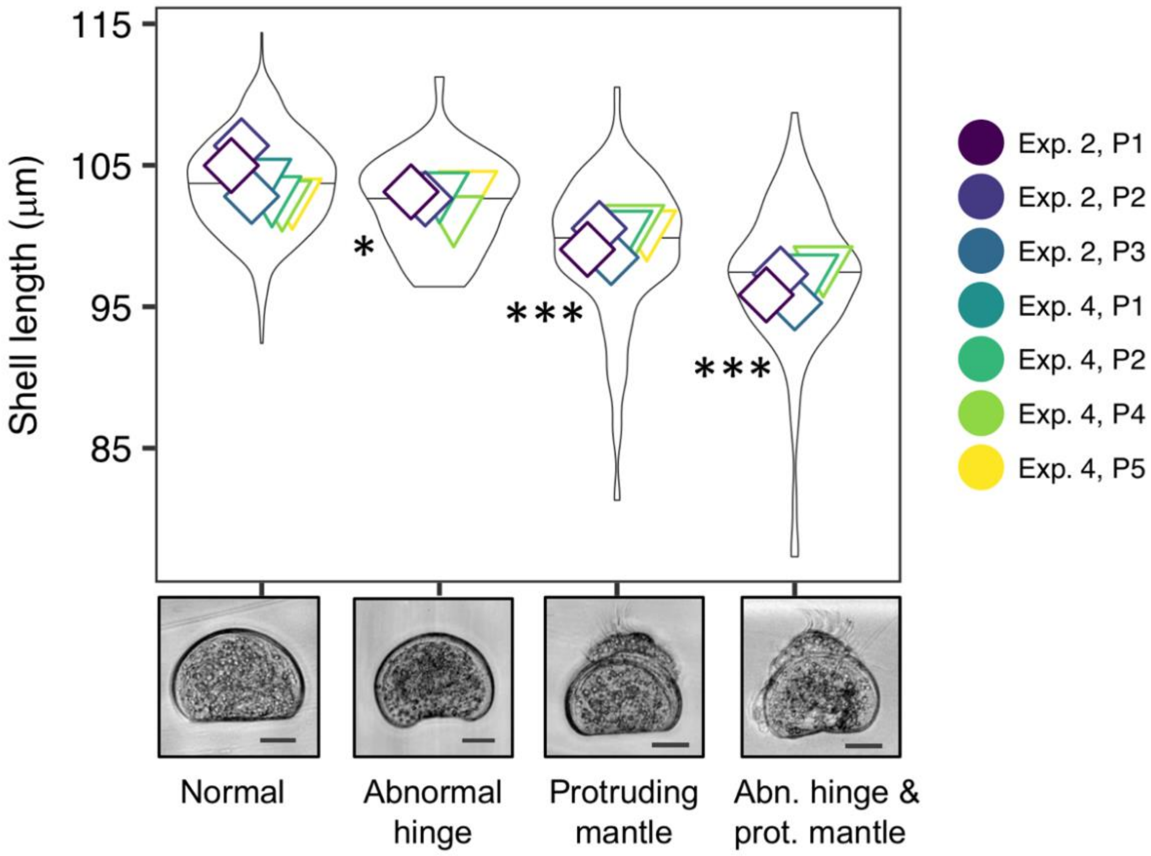

Fig. S1. Median shell size of mussel larvae by phenotype in pH_T 7.4. Size distributions of each phenotype are ordered largest to smallest from left to right. Shell length measurements were performed on larvae from unique male-female pairs (P, colored symbols) from Exp. 2 (diamond, pH_T 7.4 ---) and Exp. 4 (triangle, pH_T 7.4; see Fig. 2 for details on pH treatments). Violin plots show the size distribution across all experiments and pairs, and location of the combined median (horizontal line). Phenotype images are of a representative example; scale bar is 30 μm. Asterisk indicates significantly different median shell size relative to normal D-veligers (Table S2).

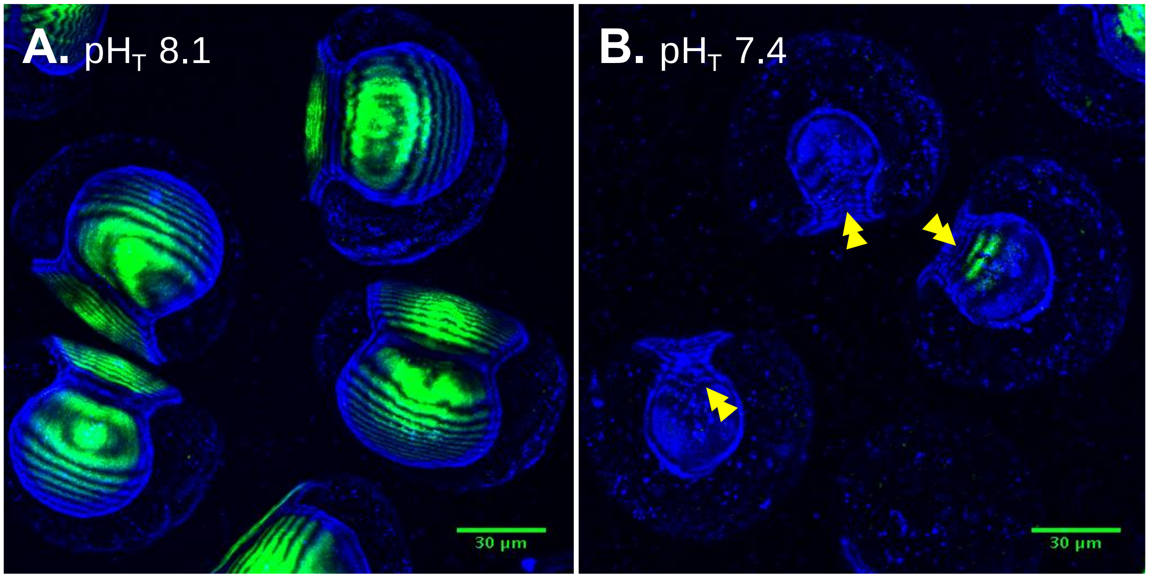

Fig. S2. Confocal images of trochophore larvae, 35 hpf, reared in pH$_T$ 8.1 and 7.4 (Exp. 4, Pair 2). Larvae in pH$_T$ 7.4 exhibit a hinge indentation (double arrowhead) in the organic matrix (blue), regardless of calcium carbonate precipitation (green). Scale bar is 30 µm.

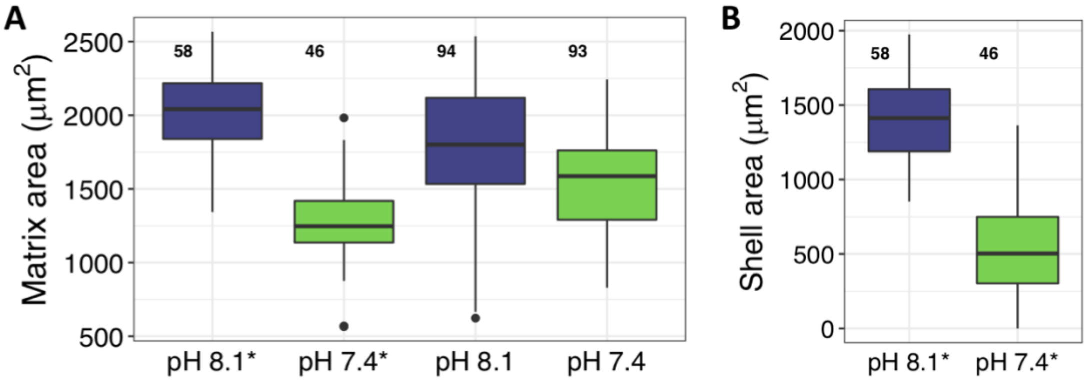

Fig. S3. Organic matrix area (A) and shell area (B) of mussel larvae at 35 hpf exposed to pH$_T$ 8.1 and pH$_T$ 7.4 in Exp. 4. Area measurements were made on one valve per larva (i.e., area of half of the organic matrix and area of one shell, per larva). Boxplots denote median, quartiles and outliers. Number of larvae measured is noted above each boxplot and represent summed measurements of larvae across parental pairs. *Cultures contained calcein dye (Pair 2 and 4): the effect of low pH on the shell matrix area appears greater in cultures with calcein (36% and 40% decrease in area) compared to cultures without calcein (12-21% area decrease). This may be due to the fact that with the addition of calcein, the high pH treatment increased slightly (0.03 units pH$_T$) and the low pH treatment decreased by 0.10 units pH$_T$, and so the magnitude of pH stress was therefore greater in cultures with calcein compared to those without.

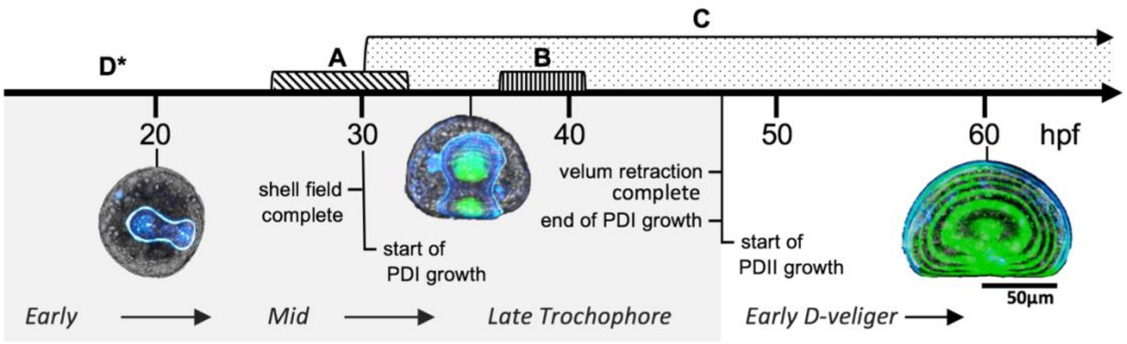

Fig. S4. Summary of the teratogenic effect of ocean acidification on mussel larvae. Developmental timeline of *M. galloprovincialis* at 14 ºC from 20 to 60 hours post-fertilization (hpf), based on observations of developmental progression and results from Exp. 1-4, showing windows of additive sensitivity to CO_2-acidified seawater (brackets above the timeline, bold letters). Abnormal shell field development from exposure around 30 hpf (**A**, diagonal hash marks) correlates to the proportion of abnormal hinge phenotypes in D-veligers. Protruding mantle D-veliger phenotypes appear to arise from exposure around 40 hpf (**B**, vertical hash marks), a period prior to the completion of velum retraction and start of PD II growth (~47 hpf). Shell growth (**C**, dotted), which starts around 30 hpf but can be delayed in low pH conditions, responds instantaneously to seawater chemistry such that mean conditions drive growth regardless of variability regimes. Timing of exposure that causes delayed shell field development (Exp. 4) cannot be identified with the data collected but likely occurs during the early trochophore stage (**D***, see text for details). Confocal images of representative larvae depict an early-mid trochophore at 20 hpf, a late trochophore at 35 hpf, and an early D-veliger at 60 hpf (same scale, organic matrix is stained blue, calcified structures are stained green, ring pattern on D-veliger is an artifact of imaging).

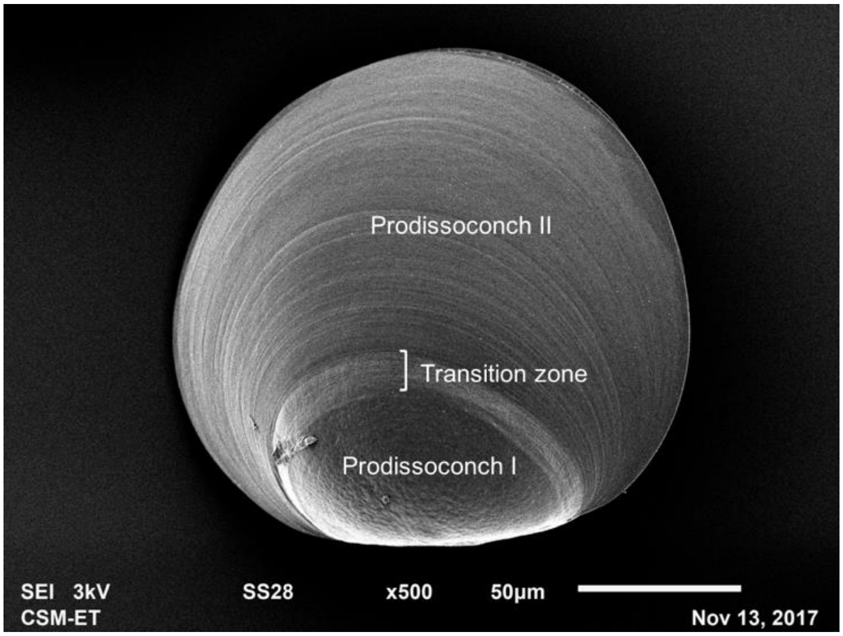

Fig. S5. SEM image of a 22-day old mussel larva in Exp.1. The transition from the first (Prodissoconch I) to second (Prodissoconch II) larval shell is marked. Hinge abnormalities are unidentifiable with certainty at this stage, due to the curvature of the shell.

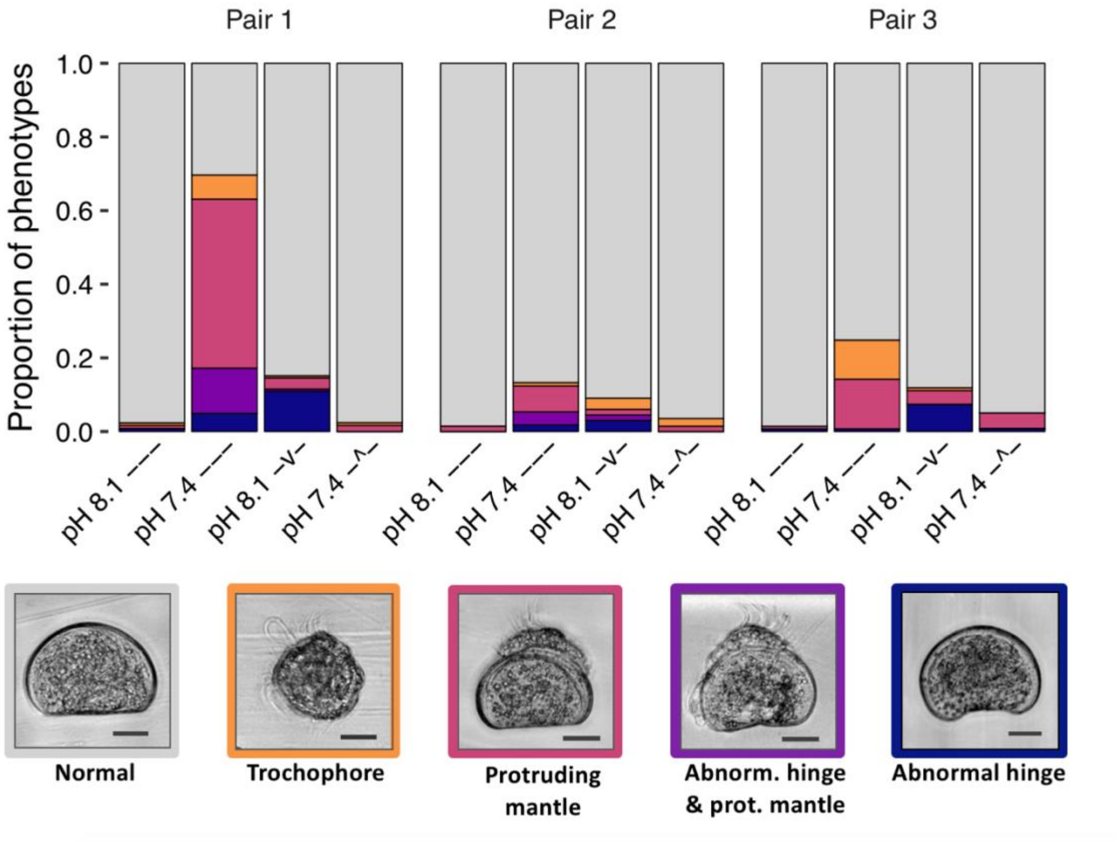

Fig. S6. Larval phenotypes from three unique parental pairs in stable and variable pH_T treatments in Exp. 2. Proportions of specific phenotypes were calculated from at least 100 observations per treatment. Color of scoring categories follows the border color of representative phenotype images (scale bar is 30 µm). See Fig. 1 in the main text for treatment labels.

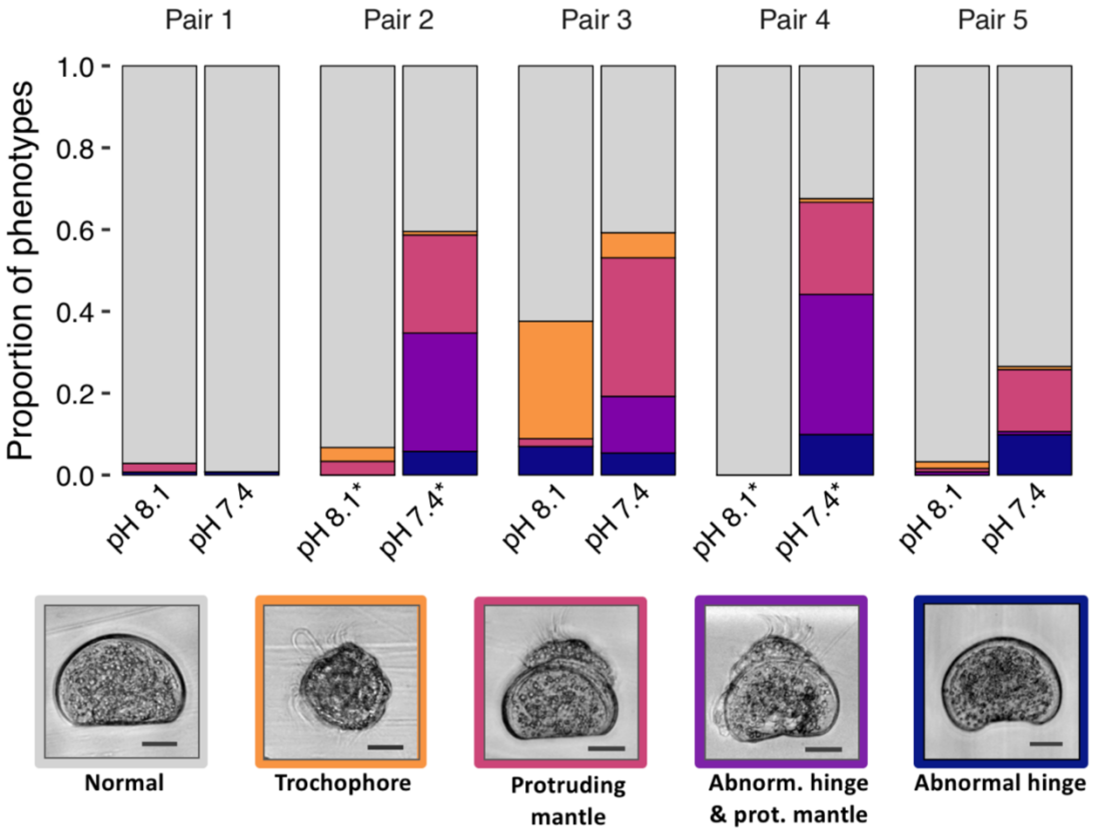

Fig. S7. Larval phenotypes from five unique parental pairs in pH_T 8.1 and pH_T 7.4 in Exp. 4. Pair 1 exhibits pH resistance with normal development in pH_T 7.4. Proportions of specific phenotypes were calculated from at least 100 observations per treatment. Color of scoring categories follows the border color of representative phenotype images (scale bar is 30 µm). *Cultures contained calcein dye (Pair 2 and 4).

Table S1. Treatment conditions for Exp. 1-4. Treatment codes follow those described in Fig. 1. Mean ± SD (*N*) is listed for non-varying parameters, and mean temperature was calculated from Durafets (5 min frequency). SIR = $[HCO_3^-]/[H^+]$

Exp.	Treatment		pH_T	Ω_a*	SIR* (mol/µmol)	pCO_2* (µatm)	T (ºC)	Salinity	AT (µmol/kg)
1	pH 7.8---	min.	7.78	1.60	0.14	784	14.4 ± 0	38.2 ± 0.2 (10)	2556 ± 11 (10)
		max.	7.82	1.77	0.15	886			
1	pH 7.8 +/-0.2	min.	7.64	1.20	0.1	436	14.3 ± 0	38.1 ± 0.1 (4)	2561 ± 2 (4)
		max.	8.05	2.75	0.24	1252			
1	pH 7.8 +/-0.4	min.	7.39	0.69	0.06	320	14.3 ± 0	38.2 ± 0.2 (10)	2556 ± 9 (10)
		max.	8.16	3.38	0.29	2322			
1	pH 7.8 -/+0.4	min.	7.37	0.67	0.06	311	14.3 ± 0	38.3 ± 0.2 (10)	2558 ± 3 (9)
		max.	8.17	3.45	0.3	2432			
2	pH 8.1 ---	min.	8.09	3.00	0.26	364	14.3 ± 0	38.5 ± 0.1 (2)	2560 ± 2 (2)
		max.	8.11	3.12	0.27	386			
2	pH 7.4 ---	min.	7.40	0.72	0.06	2204	14.4 ± 0	38.5 ± 0.1 (2)	2567 ± 7 (2)
		max.	7.41	0.73	0.06	2253			
2	pH 8.1 -v-	min.	7.26	0.53	0.04	350	14.4 ± 0	38.5 ± 0.1 (2)	2559 ± 1 (2)
		max.	8.13	3.20	0.27	3147			
2	pH 7.4 -^-	min.	7.39	0.70	0.06	320	14.3 ± 0	38.5 ± 0.1 (2)	2559 ± 1 (2)
		max.	8.16	3.39	0.29	2295			
3	pH 8.1 ---	min.	8.09	2.99	0.26	373	14.4 ± 0	38.4 ± 0.1 (4)	2564 ± 12 (4)
		max.	8.11	3.08	0.26	389			
3	pH 7.4 ---	min.	7.40	0.72	0.06	2081	14.4 ± 0	38.4 ± 0.1 (4)	2560 ± 5 (4)
		max.	7.43	0.77	0.07	2242			
3	pH 8.1 -v-	min.	7.25	0.52	0.04	352	14.4 ± 0	38.4 ± 0.1 (4)	2558 ± 7 (4)
		max.	8.13	3.18	0.27	3193			
3	pH 7.4 -^-	min.	7.40	0.72	0.06	421	14.3 ± 0	38.4 ± 0.1 (4)	2560 ± 6 (4)
		max.	8.06	2.82	0.24	2226			
4	pH 8.1	min.	8.08	2.93	0.25	363	14.2 ± 0.1	38.5 ± 0.1 (2)	2561 ± 1 (2)
		max.	8.12	3.11	0.27	396			
4	pH 7.4	min.	7.38	0.68	0.06	1087	14.2 ± 0.1	38.5 ± 0 (2)	2563 ± 1 (2)
		max.	7.7	1.35	0.12	2356			

*Parameters calculated from pH_T using mean temperature and header tank salinity and A_T, per experiment, per treatment.

†Excludes cultures with calcein dye (see Material and Methods). Salinity and A_T values represent the mean of two header tanks at the start of the experiment.

Table S2. Linear quantile mixed model results for shell size of D-veliger phenotypes reared in pH_T 7.4 (Exp. 2 and 4) over three percentiles (median, 25th, and 75th). Significant *p*-value indicates size differs from normal D-veligers based on alpha 0.05 (*), 0.001 (**), or < 0.0001(***). ± SE

**Phenotype**	**Median (µm)**	**25th (µm)**	**75th (µm)**
Normal	104.1 ± 0.5	102.0 ± 1.1	105.9 ± 1.4
Abnormal hinge	102.8 ± 0.5*	100.5 ± 0.3***	104.8 ± 0.3**
Protruding mantle	100.0 ± 0.6***	97.4 ± 0.4***	102.6 ± 0.6***
Ab. hinge & prot. mantle	97.5 ± 0.7***	94.9 ± 0.5***	100.5 ± 1.2***

Table S3. Pairwise comparisons of treatment effects on proportion of **normal D-veliger** larvae in Exp. 1 (mixed model results revealed a significant effect of treatment on normal D-veliger development in Exp. 1: χ^2 = 128.17, *df* = 3, *p*<.0001). *indicates significance at alpha 0.05, following a Bonferroni correction for 6 comparisons

Contrast	z ratio	*p*-value
pH 7.8---:pH 7.8±0.2	3.269	0.0065*
pH 7.8---:pH 7.8+/-0.4	5.895	<.0001*
pH 7.8---:pH 7.8-/+0.4	7.731	<.0001*
pH 7.8±0.2:pH 7.8+/-0.4	4.028	0.0003*
pH 7.8±0.2:pH 7.8-/+0.4	7.14	<.0001*
pH 7.8+/-0.4:pH 7.8-/+0.4	3.696	0.0013*

Table S4. Pairwise comparisons of treatment effects on proportion of **normal D-veliger** larvae in Exp. 2 (mixed model results revealed a significant effect of treatment on normal D-veliger development in Exp. 2: χ^2 = 243.8, *df* = 3, *p*<.0001) *indicates significance at alpha 0.05, following a Bonferroni correction for 6 comparisons

Contrast	z ratio	*p*-value
pH 8.1--- : pH 7.4---	8.978	<.0001*
pH 8.1--- : pH 8.1-v-	4.958	<.0001*
pH 8.1--- : pH 7.4-^-	1.642	0.6029
pH 7.4--- : pH 8.1-v-	-8.136	<.0001*
pH 7.4--- : pH 7.4-^-	-9.438	<.0001*
pH 8.1-v- : pH 7.4-^-	-4.042	0.0003*

Table S5. Pairwise comparisons of treatment effects on proportion of **normal D-veliger** larvae in Exp. 3 (ANOVA results revealed a significant effect of treatment on normal D-veliger development in Exp. 3: $F_{3,8}$ = 15.56, *p*=0.0011) *indicates significance at alpha 0.05, following a Bonferroni correction for 6 comparisons

Contrast	t ratio	*p*-value
pH 8.1--- : pH 7.4---	6.09	0.0018*
pH 8.1--- : pH 8.1-v-	3.153	0.0813
pH 8.1--- : pH 7.4-^-	0.498	1.00
pH 7.4--- : pH 8.1-v-	-2.865	0.126
pH 7.4--- : pH 7.4-^-	-5.47	0.0036*
pH 8.1-v- : pH 7.4-^-	-2.599	0.19

Table S6. Pairwise comparisons of treatment effects on proportion of larvae with **abnormal hinges** in Exp. 2 (mixed model results revealed a significant effect of treatment on normal D-veliger development in Exp. 2: $\mathcal{X}^2 = 66.858$, $df = 3$, $p<.0001$) *indicates significance at alpha 0.05, following a Bonferroni correction for 6 comparisons

Contrast	z ratio	*p*-value
pH 8.1--- : pH 7.4---	-3.834	0.0008*
pH 8.1--- : pH 8.1-v-	-3.898	0.0006*
pH 8.1--- : pH 7.4-^-	0.536	1.00
pH 7.4--- : pH 8.1-v-	-0.101	1.00
pH 7.4--- : pH 7.4-^-	3.409	0.0039*
pH 8.1-v- : pH 7.4-^-	3.449	0.0034*

Table S7. Pairwise comparisons of treatment effects on proportion of larvae with **abnormal hinges** in Exp. 3 (ANOVA results revealed a significant effect of treatment on normal D-veliger development in Exp. 3: $F_{3,8} = 42.41$, $p<.0001$) *indicated significance at alpha 0.05, following a Bonferroni correction for 6 comparisons

Contrast	t ratio	*p*-value
pH 8.1--- : pH 7.4---	-7.703	0.0003*
pH 8.1--- : pH 8.1-v-	-8.506	0.0002*
pH 8.1--- : pH 7.4-^-	-0.148	1.00
pH 7.4--- : pH 8.1-v-	-0.94	1.00
pH 7.4--- : pH 7.4-^-	7.405	0.0005*
pH 8.1-v- : pH 7.4-^-	8.2	0.0002*

References

1. Geraci M & Bottai M (2014) Linear quantile mixed models. *Statistics and Computing* 24(3):461-479.
2. Geraci M (2014) Linear Quantile Mixed Models: The lqmm Package for Laplace Quantile Regression. *Journal of Statistical Software* 57(13):1-29.
3. Kapsenberg L, *et al.* (2017) Advancing ocean acidification biology using Durafet® pH electrodes. *Frontiers in Marine Science* 4:321.
